# Racial Differences in Respiratory Morbidity in Late Preterm Infants: A Retrospective Cohort Study

**DOI:** 10.1177/2333794X241273151

**Published:** 2024-09-18

**Authors:** Theodore Dassios, Allan Jenkinson, Ravindra Bhat, Anne Greenough

**Affiliations:** 1 King’s College London, London, UK; 2 University of Patras, Patras, Greece; 3 King’s College Hospital NHS Foundation Trust, London, UK

**Keywords:** race, Black, White, late preterm, ventilation

## Abstract

*Objectives*. The role of race in late preterm respiratory morbidity has not been adequately described. We aimed to determine whether neonatal respiratory morbidity differs between Black and White late preterm infants. *Methods*. Single-centre retrospective cohort study at King’s College Hospital NHS Foundation Trust, London, UK of infants born at 34 to <37 weeks of gestational age. The incidence of invasive ventilation was used as the main outcome. *Results*. In the study period 354 Black and 673 White late preterm infants were admitted. Black, compared to white infants, had a lower incidence of invasive ventilation (19% vs 27%, *P* < .001) and a lower incidence of non-invasive ventilation (22% vs 34%, *P* < .001). Black infants had a shorter duration and cost of stay compared to White infants (*P* = .011 and <0.001 respectively). *Conclusion*. Black late preterm infants needed less frequently invasive and non-invasive ventilation and had a shorter duration and cost of stay compared to White late preterm infants.

## Introduction

Late preterm infants are defined as those born between 34 and <37 weeks of gestation and account for up to 75% of premature births.^1^ Although late preterms constitute a lower-risk category of infants compared to very or extremely preterm ones, they suffer from increased morbidity and exert considerable pressure on neonatal services, because of their large number and frequent admission to neonatal services.^2^ Morbidity among late preterm infants is more pronounced in the presence of other co-morbidities such as intrauterine growth retardation, lack of antenatal steroid administration and other maternal or perinatal complications.^3^

Racial disparities in neonatal respiratory outcomes have been previously described mostly in relation to extremely preterm and very preterm infants. Wallace et al^4^ in a retrospective cohort study of preterm infants from the United States described that Black Infants had a significantly lower incidence of respiratory distress syndrome and higher incidence of transient tachypnoea of the newborn compared to White or Hispanic infants, despite a similar risk for neonatal death. We have also previously described a lower incidence of respiratory distress syndrome in Black compared to White very preterm infants.^5^ The role of race, however, in respiratory morbidity in late preterm infants has been less extensively studied.

The neonatal unit at King’s College Hospital (KCH) serves a diverse community in South East London with approximately 30% of the population identifying as of Black background, one of the highest in the United Kingdom.^6^ The neonatal unit of KCH is thus a well-placed institution to delineate the contribution of race in later preterm respiratory morbidity. In this study we hypothesised that Black infants would suffer less respiratory morbidity compared to White infants. Our aim was to test this hypothesis.

## Materials and Methods

A retrospective cohort study of all admissions to the Neonatal Unit at KCH of infants born between 34 and <37 (36 completed) weeks of gestation between 1 January 2012 and 1 January 2023 (11 years) was undertaken. The starting year of 2012 was selected for purposes of data entry consistency and to reflect current neonatal practice. KCH has a tertiary neonatal unit with approximately 6000 deliveries per year and serves a diverse community of over 1 000 000 in South East London by providing all levels of neonatal care (intensive care, high dependency and special care). Infants were classified as Black or White based on parental self-identification. Infants whose parents self-identified as of Asian, mixed or unspecified ethnicity represented a smaller proportion of admissions and were excluded from analysis, due to insufficient numbers for meaningful analysis. Data were extracted from the BadgerNet Neonatal Electronic Patient Database (Clevermed, Edinburgh, UK).

The following data were collected: full course of antenatal steroids (yes/no), maternal age (years), sex (male/female), gestational age (weeks), birth weight (kg), Apgar score at 5 minutes, inborn at KCH (yes/no), admission temperature (°C), admission blood glucose (mmol/L), need for parenteral nutrition (PN) via central line (yes/no), invasive mechanical ventilation (yes/no), duration of invasive ventilation (days), non-invasive ventilation in the form of continuous positive airway pressure (CPAP) or high-flow nasal cannula (yes/no), duration of non-invasive ventilation (days), duration of supplementary oxygen (days), duration of stay (days). The total cost of stay was calculated according to level of care at 1118 GBP per day in intensive care, 791 GBP per day in high dependency and 505 GBP per day in special care.^7^ Mortality was defined as death before discharge from neonatal care. As the neonatal unit at KCH is a regional neurosurgical and surgical referral centre, a separate analysis was undertaken further excluding major congenital surgical diagnoses such as congenital diaphragmatic hernia, gastroschisis, exomphalos, oesophageal atresia, bowel atresia, Hirschsprung’s disease, congenital pulmonary airway malformation, congenital hydrocephalus, spina bifida and trisomy 21.

The study was registered with the Department of Clinical Governance of KCH. The Health Research Authority Toolkit of the National Health System, United Kingdom verified that the study would not need regulatory approval by a research ethics committee.

## Statistical Analysis

Continuous data were tested for normality with the Kolmogorov-Smirnov test and found to be non-normally distributed and were therefore presented as median and interquartile range (IQR). The primary analysis aimed to determine if there was a statistically significant difference in the incidence of invasive ventilation in Black and White infants and was undertaken using the Chi square (*x*^2^) test. The incidence of antenatal steroids, male sex, inborn and mortality were compared between Black and White infants using the *x*^2^ test. The maternal age, gestational age, birth weight, Apgar score at 5 minutes, admission temperature, admission blood glucose, duration of invasive ventilation, duration of stay and cost of stay were compared in Black and White infants using the Mann-Whitney *U* non-parametric test. Comparisons between Black and White infants were repeated in infants without major congenital anomaly and surgical diagnosis. The adjusted contribution of parental ethnic background to the incidence of ventilation was examined using binary regression analysis after adjusting for parameters that were significantly different between Black and White infants (*P* < .05) at a univariate level. Multi-collinearity among the independent variables in the regression analysis was assessed by examination of a correlation matrix for the independent variables.

Statistical analysis was performed using SPSS software, version 27.0 (SPSS Inc., Chicago, Illinois, USA).

## Results

In the study period 7414 infants of all gestations were admitted to the Neonatal Unit at KCH (674 per year), of whom 1369 (124 per year) were born between 34 and <37 weeks of gestation. The total cost of stay for all admitted infants was 106 556 002 GBP and for the late preterms was 13 818 343 GBP (13% of the cost of all admitted infants).

**Figure 1.**
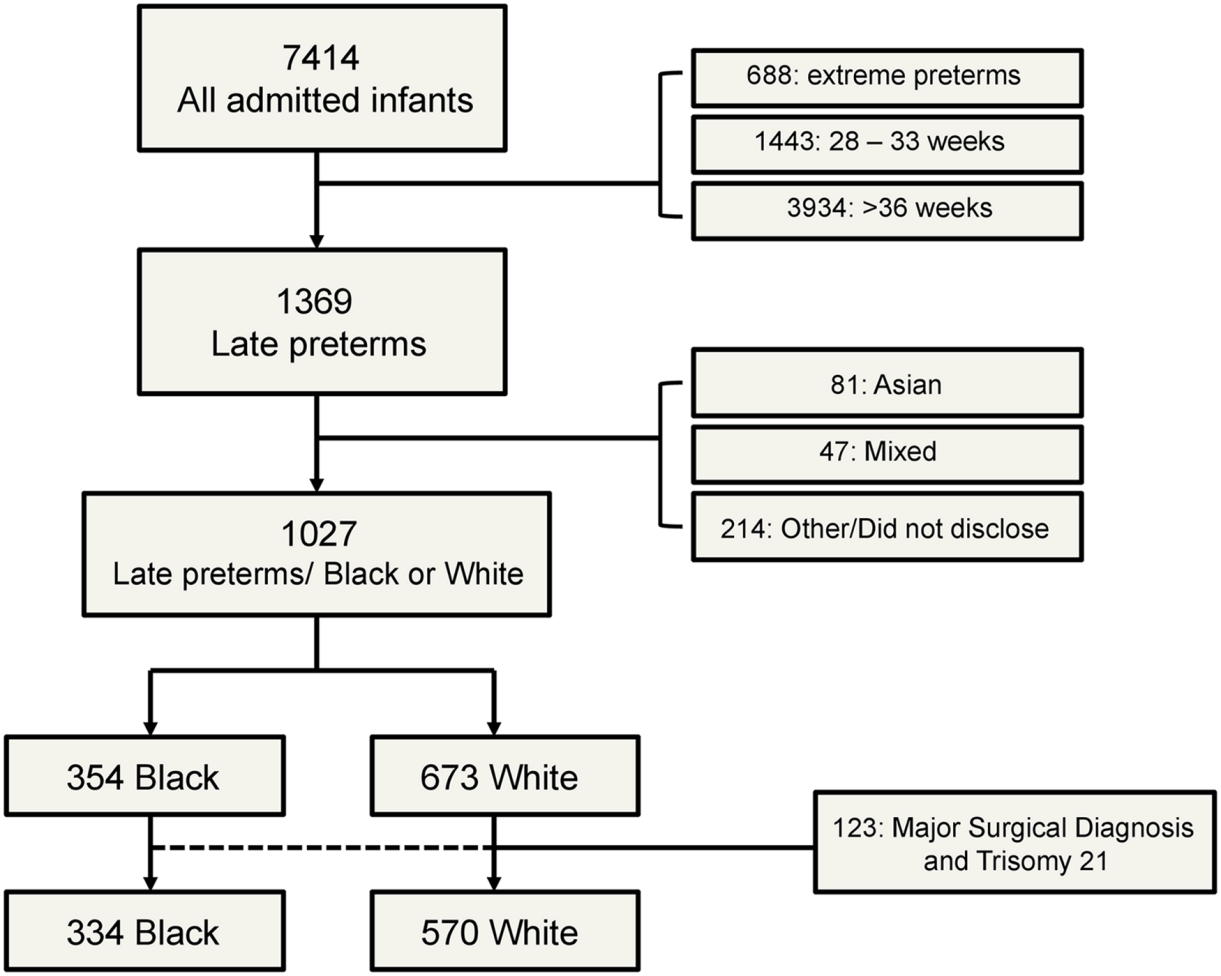
Flow diagram of the study population and included groups.

Eighty-one, 47 and 214 infants were born to parents who self-identified as Asian, Mixed or other/or did not disclose their ethnic background and were thus excluded from subsequent analysis. The final analysis included 1027 late preterm infants: 354 were Black and 673 were White (Figure 1). The characteristics of the study population in Black and White infants are presented in Table 1. Black infants were born to younger mothers (*P* = .013), were more frequently female (*P* < .001), had lower birth weight (*P* < .001), were more commonly inborn (*P* < .001) and had lower admission temperature (*P* = .002) and blood glucose (*P* = .024, Table 1). Black infants had a lower incidence of invasive ventilation (19%) compared to white infants (27%, *P* < .001) and were ventilated for shorter periods (*P* < .001). Black infants had shorter duration and cost of stay compared to White infants (*P* = .011 and <0.001 respectively, Table 1).

**Table 1.** Characteristics of the Included Infants (N = 1027). Data are Presented as Median (interquartile Range) or N (%).

		Black	White	*P*-value
		N = 354	N = 673	
Antenatal	Antenatal steroids	225 (64)	423 (63)	.808
	Maternal age	32 (28-36)	33 (30-37)	.013
Birth	Male sex	169 (48)	394 (59)	<.001
	Gestational age (weeks)	35.57 (34.57-36.29)	35.57 (34.57-36.29)	.959
	Birth weight (g)	2200 (1900-2535)	2380 (1990-2735)	<.001
	Apgar at 5 minutes	10 (9-10)	10 (9-10)	.501
	Inborn at KCH	341(96)	590 (88)	<.001
Admission	Admission temperature	36.7 (36.4-36.9)	36.7 (36.5-37.0)	.002
	Admission glucose	2.5 (1.9-3.5)	2.7 (3.0-3.9)	.024
Neonatal unit	Need for PN	63 (18)	180 (27)	.001
	Invasive ventilation	67 (19)	211 (31)	<.001
	Non-invasive ventilation	79 (22)	227 (34)	<.001
	Duration of ventilation	0 (0-0)	0 (0-1)	<.001
	Duration of oxygen	0 (0-3)	1 (0-3)	<.001
Discharge	Died	3 (1)	11 (2)	.301
	Duration of stay (days)	6 (3-13)	8 (4-15)	.011
	Cost of stay (GBP)	3281 (1800-7643)	5050 (2020-9593)	<.001

Abbreviations: KCH, King’s College Hospital; PN, parenteral nutrition; NIV, non-invasive ventilation; GBP, British pound.

Twenty Black and 103 White infants had a major surgical diagnosis or Trisomy 21 (Figure 1). These exclusion diagnoses were more common in White compared to Black infants (*P* < .001). Excluding major surgical pathology and trisomy 21, Black infants were more frequently female (*P* = .004), had lower birth weight (*P* < .001) and admission temperature (*P* < .001, Table 2). The incidence of invasive ventilation was significantly lower in Black (15%) compared to White infants (22%, *P* = .013) (Table 2). Following binary regression analysis with the incidence of ventilation as the outcome parameter, Black ethnic background was significantly related to a decreased incidence of invasive ventilation (Odds Ratio: 0.879, 95% CI: 0.778-0.994, adjusted *P* = .040) after adjusting for birth weight (adjusted *P* = .006), male sex (adjusted *P* = .36) and admission temperature (adjusted *P* = .043).

**Table 2.** Characteristics of the Study Population Excluding Infants With a Major Surgical Diagnosis (N = 904). Data are Presented as Median (interquartile Range) or N (%).

	Black	White	*P*-value
	N = 334	N = 570	
Antenatal steroids	212 (63)	354 (62)	.683
Male sex	164 (49)	336 (59)	.004
Gestational age (weeks)	35.43 (34.57-36.29)	35.57 (34.57-36.29)	.878
Birth weight (g)	2200 (1900-2535)	2400 (1995-2735)	<.001
Inborn at KCH	323(97)	513 (90)	<.001
Admission temperature	36.7 (36.4-36.9)	36.7 (36.5-37.0)	.001
Admission glucose	2.5 (1.9-3.5)	2.7 (2.0-3.9)	.071
Invasive ventilation	50 (15)	124 (22)	.013
Died	1 (0.3)	5 (0.9)	.302
Cost of stay (GBP)	3030 (1565-6850)	4040 (2020-7575)	.106

Abbreviations: KCH, King’s College Hospital; GBP, British pound.

## Discussion

We have demonstrated that Black late preterm infants needed less frequently invasive and non-invasive ventilation and had a shorter duration and cost of stay compared to White late preterm infants. To our knowledge, there is only one other study that specifically investigated respiratory morbidity in late prematurity according to race/ethnic background. In a group of 2331 women from the US which included a group of Black (26.9%) and White (57.1%) mothers, there was no overall difference in respiratory morbidity between racial groups. Their study included a secondary outcome of ‘severe respiratory morbidity’ which included invasive mechanical ventilation, CPAP or high-flow nasal cannula for at least 12 hours, and this outcome was less common in Black compared to White infants.^8^ That study might not be directly applicable to the UK due to healthcare system differences and differences in the ethnic background of the mothers and infants included in the Black group.^9^ Our study’s results, however, are in agreement with the aforementioned study and complement the literature by reporting similar outcomes in a British cohort cared by a healthcare system with universal free access to maternity and other healthcare services.

Although mortality would be rare in late preterm infants, our results agree with previous studies that did not report a difference in survival among different ethnic groups. Wallace et al^4^ in a large retrospective cohort of preterm infants reported that infants were at a similar risk for neonatal death, regardless of race. We have also previously reported that in our population of all admitted infants irrespective of gestation, socioeconomic status, gestational age and admission hypothermia were independently associated with neonatal mortality, but racial background was not.^10^ We have also reported that although adult studies have highlighted the presence of a possible ‘occult hypoxaemia’ in Black infants,^11^ ethnic differences were not present in saturation monitoring in preterm infants born at less than 32 weeks of gestation, and thus could not explain differences in respiratory outcomes.^6^

The precise mechanisms by which Black infants were relatively protected compared to White infants from respiratory morbidity have not been completely elucidated. One possible mechanism might be that antenatal corticosteroids act faster and have a shorter bioavailability in mothers of Black ethnicity. Corticosteroid metabolism is controlled by the enzymes of the cytochrome p450^12^ which most African-Americans express, compared to only 10% of Caucasians.^13^ This finding might also be relevant in our population as the incidence of antenatal corticosteroids administration was not significantly different between the mothers of Black compared to White infants. Another possible mechanism for the relative protection of Black preterm infants from respiratory morbidity is the earlier foetal lung maturity seen in Black infants and qualitative differences in surfactant between racial groups. Foetal lung maturity has been ascertained from amniotic fluid sphingomyelin/lecithin ratio, and Black infants had higher ratios at earlier gestational ages compared to White infants.^14^

Interestingly, Black infants exhibited a relative advantage in respiratory morbidity, despite some unfavourable demographics, such as a lower birth weight, higher incidence of admission hypothermia and lower admission blood glucose which perhaps can be seen as a proxy for other co-morbidities which are more frequent in mothers of Black infants such as pregnancy induced hypertension.^15^

In our study we used the incidence of invasive ventilation as the main index of respiratory morbidity. Other studies have utilised more specific diagnoses such a respiratory distress syndrome and transient tachypnoea of the newborn.^4^ It is sometimes difficult, though, to completely separate those diagnoses in clinical practice or deduct the contribution of less well-defined entities such as pulmonary hypoplasia and suspected pulmonary or systemic infection, which cannot be robustly proven at a population level. We chose, thus, a binary measure of respiratory morbidity that would be less subjective to operator-dependent interpretation error. Arguably, in our study we only included admitted infants and a number of late preterm infants would not require admission to a neonatal unit. Some minor morbidity, such a longer stay in a postnatal ward, would thus not be captured by our study but our main outcome which was invasive ventilation could not possibly occur outside a neonatal unit.

Our study has strengths and some limitations. In our unit, the high percentage of the population self-identifying as Black is one of the highest in the UK and we included a large number of late preterms. Although we did not conduct a formal sample size calculation, we extended our study period to include the largest possible number of infants and reached statistically significant results. Since the study was a retrospective one, it was not possible to exclude the inherent bias of retrospective studies. A prospective study, however, assigning infants to different intervention groups according to ethnic background would not be possible. We did not account for socioeconomic differences as indeed these might influence prematurity-related outcomes. The demographics and economic environment in South East London have changed, however, over the recent years secondary to gentrification and an index of multiple deprivation corresponding to one specific address in 2012 would not reflect the same level of socioeconomic standing in 2023.^16^

## Conclusions

We have demonstrated that Black late preterm infants needed less frequently invasive and non-invasive ventilation and had a shorter duration and cost of stay compared to White late preterm infants.
